# Carbonate buffer mixture and fecal microbiota transplantation hold promising therapeutic effects on oligofructose-induced diarrhea in horses

**DOI:** 10.3389/fvets.2024.1388227

**Published:** 2024-04-22

**Authors:** Maimaiti Tuniyazi, Ruibo Tang, Xiaoyu Hu, Yunhe Fu, Naisheng Zhang

**Affiliations:** College of Veterinary Medicine, Jilin University, Changchun, China

**Keywords:** diarrhea, oligofructose, gut microbiota, CBM, FMT, horse

## Abstract

Diarrhea is a common gastrointestinal disorder in horses, with diet-induced diarrhea being an emerging challenge. This study aimed to investigate the gut microbiota differences in healthy and diet-induced diarrheic horses and evaluate the effectiveness of fecal microbiota transplantation (FMT) and carbonate buffer mixture (CBM) as potential therapeutic approaches. Twenty healthy horses were included in the study, with four groups: Control, Diarrhea, CBM, and FMT. Diarrhea was induced using oligofructose, and fecal samples were collected for microbiota analysis. FMT and CBM treatments were administered orally using donor fecal matter, and formula mixture, respectively. Clinical parameters, serum levels, intestinal tissue histopathology, and fecal microbiota profiles were evaluated. The results showed that diarrhea induction disbalanced the gut microbiota with decreased diversity and richness, affected clinical parameters including elevated body temperature and diarrhea score, and decreased fecal pH, increased inflammatory responses such as increased serum LPS, IL-17A, lactic acid and total protein, and caused damage in the colon tissue. CBM and FMT treatments altered the gut microbiota composition, restoring it towards a healthier profile compared to diarrheic, restored the gut microbiota composition to healthier states, improved clinical symptoms including decreased body temperature and diarrhea score, and increased fecal pH, decreased inflammatory responses such as increased serum LPS, IL-17A, lactic acid and total protein, and repaired tissue damage. CBM and FMT Spearman correlation analysis identified specific bacterial taxa associated with host parameters and inflammation. FMT and CBM treatments showed promising therapeutic effects in managing oligofructose-induced diarrhea in horses. The findings provide valuable insights into the management and treatment of diarrhea in horses and suggest the potential of combined CBM and FMT approaches for optimal therapeutic outcomes.

## 1 Introduction

Diarrhea is a common and substantial gastrointestinal disorder in horses, associated with considerable morbidity and economic losses within the equine industry (1, 2). Diarrhea continues to be a leading cause of critical illness in horses, demonstrating an estimated fatality rate of 25.4 to 35% (3, 4). The effectiveness of antibiotic therapy in the treatment of diarrheic horses is frequently limited and can even exacerbate the condition. This is due to the unintended consequence of eradicating beneficial commensal bacteria in the gut, thereby creating an environment conducive for the proliferation of pathogenic species. The inadequacy of targeted and efficacious therapies for equine diarrhea poses challenges for clinicians in promptly reversing fluid losses and mitigating systemic inflammation associated with the disease. These factors contribute to increased complication rates and necessitate prolonged hospital stays with intensive care.

Although diarrhea has multifactorial causes, emerging research indicates that an imbalanced gut microbiota, known as dysbiosis, plays a pivotal role in its development. Indeed, the composition of the gut microbiota is a complex ecosystem of microorganisms that interact with the host and contribute to various physiological processes, including nutrient digestion and metabolism, immune modulation and maintaining gastrointestinal health (5–7). Disruptions in the gut microbiota composition can lead to dysbiosis, characterized by an imbalance of microbial populations and potential pathogenic overgrowth (8, 9), which has been associated with gastrointestinal disorders, including diarrhea (10). Therefore, understanding the impact of gut microbiota during the occurrence and treatment of diarrhea in horses is essential for developing effective gut microbiota manipulation-based management strategies and promoting equine well-being.

Oligofructose has been extensively used to induce a controlled diarrhea model in horses, closely mimicking gut microbiota disturbances observed in clinical cases (11, 12). This model allows us to investigate the alternations in gut microbiota associated with diarrhea in a controlled manner and evaluate potential interventions for modulating the gut microbiota.

Fecal microbiota transplantation (FMT) is a therapeutic procedure aimed at restoring the healthy composition of gut microbes that may have been disturbed by antibiotic use, pathogenic invasion, or dietary changes (13). The origins of FMT can be traced back to the 4th century AD, and since its approval by the US Food and Drug Administration (FDA) in 2013 for the treatment of *Clostridium difficile* infection (CDI) in humans (14), it has garnered significant attention and research interest (15). In the case of horses, there exists a limited exploration regarding the effectiveness of FMT, with only a few available studies presenting their outcomes. These studies have revealed mixed results, pointing towards some positive effects of FMT in treating certain intestinal disorders such as colitis and diarrhea (16–18). However, it is important to note that these studies were not case-controlled and involved horses from various locations with differing health statuses serving as controls. Conversely, other studies have demonstrated no discernible impact of FMT on gastrointestinal issues in horses (19–22). Consequently, it is crucial to approach these findings with caution and acknowledge the necessity for further research to enhance the efficacy of FMT in equine care.

Carbonate buffer mixture (CBM) is recognized for its exceptional buffering capability that makes it an effective regulator of pH levels in the gastrointestinal tract (23). The pH levels within the gut are critical in preserving the balance of the microbiota ecosystem (24). Imbalances or disruptions in gut pH could create conditions that encourage the growth of detrimental bacteria or inhibit the proliferation of beneficial ones (25). CBM can aid in stabilizing and maintaining optimal pH levels, thereby fostering a more conducive environment for the growth and activity of beneficial gut bacteria (26). Studies have underscored the potential influence of CBM on the composition of gut microbiota (27). By modulating gut pH, CBM could potentially have a positive impact on the diversity and abundance of microbial populations, thus encouraging a healthier microbiota profile (28). This, in turn, can contribute to improved overall gastrointestinal function (29).

The aims of this study were: to investigate the gut microbiota differences in groups of healthy and diet-induced diarrhea ponies, and to use 16S *rRNA* gene sequencing of fecal samples to determine the alterations to the fecal microbiota associated with diarrhea; to examine the effectiveness of FMT as a therapeutic method for treating Oligofructose-induced diarrhea in horses as well as its impact on the gut microbiota; to investigate the role of CBM in treating gut microbiota dysbiosis-related diarrhea in horse, regulating pH levels in the gastrointestinal tract and its impact on the composition of gut microbiota.

## 2 Materials and methods

### 2.1 Ethical statement

The full proposal was reviewed by the Institutional Animal Care and Use Committee (IACUC) of Jilin University ethics committee, which approved the animal care and use permit license. All methods were performed in accordance with the relevant guidelines and regulations of IACUC.

### 2.2 Animals and diet management

Twenty healthy horses (10 stallions and 10 mares, 3 years old, weight: 308 ± 26 kg) were purchased by the authors from a horse ranch and were born during the foaling season of 2020. They have been raised under consistent and controlled conditions since birth, with the specific intention of using them for meat supply purposes. The selection of the 20 horses for this study was based on thorough evaluation of their medical records, which included detailed information regarding any previous diseases, treatments, as well as veterinary clinical examinations. Only horses with a clean bill of health, i.e., those that did not exhibit any digestive disorders, had no recent exposure to antibiotics or anthelmintic treatments, and had not undergone long-distance transportation within the past 3 months, were included in the study.

The diet has a significant impact on the gut microbiota composition in horses (30). While the selected horses were managed under similar conditions, we implemented additional measures to establish a highly controlled feeding environment. Therefore, we isolated the chosen horses and provided them with a locally available forage-based diet (Table 1) for 2 months. Each horse was fed a daily maintenance ration equivalent to 2% of their body weight on a dry matter (DM) basis (31). The horses had unrestricted access to water, and no additional dietary supplements were administered.

**Table 1:** Chemical composition of the horse diet.

Name	Percentage
DM^a^ (%NM^b^)	90.75
CP^c^ (%DM)	8.12
CF^d^ (%DM)	1.67
Ash (%DM)	4.16
NDF^e^ (%DM)	43.72
ADF^f^ (%DM)	32.95
Ca^g^ (%DM)	0.17
P^h^ (%DM)	0.05

a Dry matter.

b Natural matter.

c Crude protein.

d Crude fat.

e Neutral detergent fiber.

f Acid detergent fiber.

g Calcium.

h Phosphorus.

### 2.3 Induction of diarrhea

**Figure 1:**
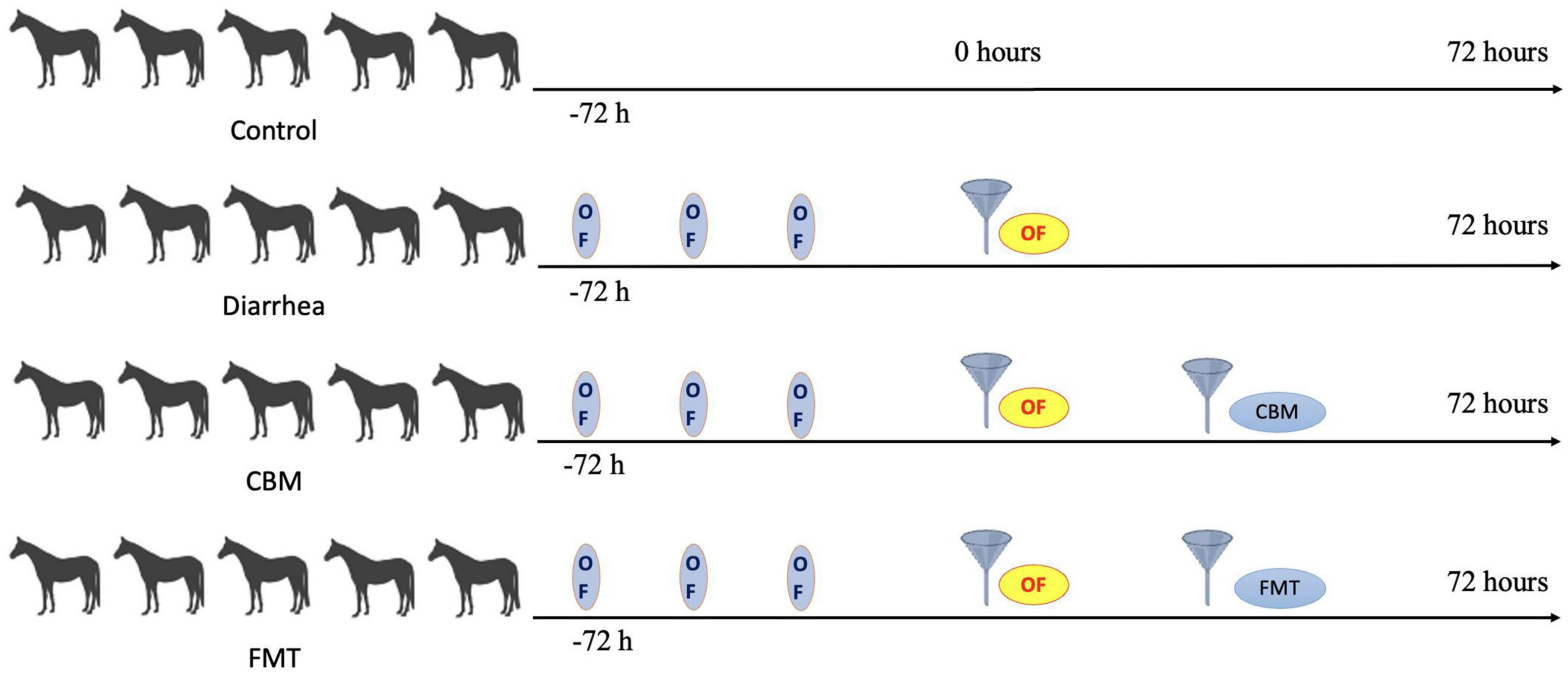
Illustration of timeline of this study.

The oligofructose-induced diarrhea was accomplished with the procedure described in our previous study (9). In brief, after 2 months of dietary acclimatization, horses were randomly assigned into four different groups: Control (*n* = 5), Diarrhea (*n* = 5), FMT (*n* = 5), and CBM (*n* = 5). Three days before the induction of diarrhea, the horses in Diarrhea, FMT, and CBM group were fed with 1 g/kg body weight of oligofructose mixed in diet to accommodate the gut microbiota. After that, diarrhea model was induced by 10 g/kg body weight of oligofructose. More specifically, the oligofructose was dissolved in 10 L of lukewarm water and administrated to the horses using a nasogastric tube. Fecal samples were collected via rectum at the same time to ensure comparability among groups and immediately stored at −80°C until microbiota analysis. Fecal pH, body temperature, and diarrhea scoring were detected at 4 h intervals. Diarrhea scoring in horses was conducted using a previously established scale (18) ranging from 0 to 5: 0—normal: firm but moist balls of manure that retain their shape; 1—soft-formed: balls of manure that appear slightly softer and lose their form upon reaching the ground; 2—pudding-consistency: manure with a pudding-like consistency that still maintains some shape; 3—pudding-consistency: manure with a pudding-like consistency that spreads out upon reaching the ground; 4—watery manure: watery manure with some remaining formed, recognizable pieces; 5—watery manure: watery manure lacking any clearly formed or recognizable pieces. A brief experimental timeline was shown in Figure 1.

### 2.4 Fecal microbiota transplantation

Conforming to the methodologies outlined in previous studies (16, 18–20), fresh fecal samples were obtained per rectum from Control horses. For each horse in the FMT group, after the successful induction of diarrhea (approximately 20 h after oligofructose administration), approximately 3 kg of mixed donors’ fecal matter was thoroughly mixed with 5 L of lukewarm water. The mixture, then, was subjected to a two step-filtration process prior to administration, which was done to ensure that only the desired microbial components were introduced into the recipient horses, while larger particulate matter was effectively removed. In the first step, the solution was poured through a coarse sieve. This primary filtration step served to remove any large particulates that could potentially cause blockage in the nasogastric tube during administration. Following this, the solution underwent a secondary filtration. This step was crucial in removing smaller, yet still potentially harmful, particulates from the solution, thereby facilitating a smooth administration and ensuring the safety of the recipient horses.

### 2.5 Carbonate buffer mixture treatment

The specific chemical composition of carbonate buffer mixture (CBM) is detailed in Table 2. For each equine participant in the CBM group, following the successful induction of diarrhea (approximately 20 h after oligofructose administration), CBM was prepared. This was done by dissolving the required amount of the mixture in 10 L of lukewarm water, ensuring through dissolution and homogeneity of the solution. The prepared CBM solution was then administrated to the horses via a nasogastric tube, ensuring direct and efficient delivery.

**Table 2:** Chemical composition of the carbonate buffer mixture (CBM).

Name	Amount (g)	Percentage (%)
Na_2_CO_3_	50	8.5
NaHCO_3_	420	71.2
KCl	20	3.4
NaCl	100	16.9

### 2.6 LPS concentration detection

Blood samples were obtained from the jugular vein of the horses and centrifuged at 3,000 g for 30 min at 4°C. After centrifugation, the supernatants (serum) were carefully transferred into sterile and dehydrogenated glass tubes. To determine the concentration of LPS, a chromogenic endpoint assay was utilized. The assay was performed following the manufacturer’s instructions (Chinese Horseshoe Crab Reagent Manufactory Co., Ltd., Xiamen, China) and had a minimum detection limit of 1 ng/mL.

### 2.7 Lactic acid, IL-17A, and total protein concentration detection

Blood samples were collected and centrifuged at 3,000 g for 30 min at 4°C, and the serum were collected to detect the concentration of lactic acid and IL-17A using ElISA kits according to the manufacturer’s instructions (MLBIO Biotechnology Co. Ltd., Shanghai, Suzhou, China) and had a minimum detection limit of 1 μg/mL and 1 pg/mL, respectively. Total protein concentration was measured using IDEXX VetAutoread (IDEXX US) and had a minimum detection limit of 1 g/L.

### 2.8 Euthanasia

To conduct the necessary pathological assessments, the horses were humanely euthanized. Euthanasia was performed following a well-established protocol, utilizing a Xylazine-Ketamine composition (IS Abundant Pharmaceutical Co. Ltd., Lanzhou China) at a specific ratio of 1:5 [0.1 mL/kg (9)]. The pre-euthanasia drug was administered intravenously into the jugular vein at a controlled rate of 0.5–1 mL/s. Subsequently, sodium pentobarbital (Feilong Pharmaceutical CO., LTD, Heilongjiang China) was injected through the jugular vein at a dosage of 0.1 mL/kg. To ensure the efficacy of euthanasia, a veterinary specialist confirmed the animal’s unconsciousness, lack of sensitivity to pain, and absence of a heartbeat. This confirmation involved a needle prick test on the surface of the ears, ensuring the absence of any response indicative of pain or consciousness.

### 2.9 Hematoxylin and eosin staining of intestinal tissue

The horses utilized in this study were primarily intended for meat production, hence, at the end of the experiment, they were humanely euthanized by the ranch owner. Post-mortem, intestinal tissues were promptly harvested within an hour. From each horse, a 10 × 10 × 0.5 mm tissue sample (sourced from the colon) was dissected and segmented into 55 mm-square fragments, which were then preserved in 4% formalin for a period between 24 to 72 h. This was followed by routine processing and embedding in paraffin wax. A standardized procedure was followed to ensure that all tissue samples were collected from the same anatomical location across all horses. Hematoxylin and Eosin (H&E) staining methods were employed, which were subsequently analyzed under a standard light microscope. These H&E-stained slides served dual purposes: the detection of intestinal lesions and the performance of morphometric analysis. All visual observations were digitally captured using a specialized image capture software coupled with a microscope-integrated camera (Olympus, Japan).

### 2.10 Total bacterial DNA extraction and Illumina NovaSeq sequencing

Fecal samples from horses (*n* = 20, 5 horses from each group) were used for bacterial DNA extraction and subsequent microbial analysis. DNA extraction from equine fecal samples was carried out using the CTAB method as per the manufacturer’s protocol. The CTAB method effectively enables the recovery of DNA from trace amounts of the sample and has been validated for the preparation of DNA from diverse bacterial species. Blank samples were treated with nuclear-free water. The extracted total DNA was eluted in 50 μL of Elution buffer and stored at −80°C until further analysis.

For amplification of the V3–V4 region of the 16S rDNA genes, we employed the primer set 314F (5′-CCTACGGGNGGCWGCAG-3′) and 805R (5′-GACTACHVGGGTATCTAATCC-3′), with barcodes attached to the 5′ ends of the primers. The primers were designed to accommodate the sequencing universal primers as well. PCR amplification reactions were performed in a total volume of 25 μL, containing 25 ng of template DNA, 12.5 μL of PCR Premix, 2.5 μL of each primer, and PCR-grade water for volume adjustment. The PCR conditions for amplifying prokaryotic 16S fragments involved an initial denaturation at 98°C for 30 s, followed by 32 cycles of denaturation at 98°C for 10 s, annealing at 54°C for 30 s, extension at 72°C for 45 s, and a final extension at 72°C for 10 min. The amplification products were confirmed using 2% agarose gel electrophoresis. As a negative control, ultrapure water was included throughout the DNA extraction process in place of a sample solution to rule out false-positive PCR results. The PCR products were purifyied by AMPure XT beads (Beckman Coulter Genomics, Danvers, MA, United States) and quantified by Qubit (Invitrogen, United States). The amplicon pools were prepared for sequencing and the size and quantity of the amplicon library were assessed on Agilent 2100 Bioanalyzer (Agilent, United States) and with the Library Quantification Kit for Illumina (Kapa Biosciences, Woburn, MA, United States), respectively. The libraries were sequenced on NovaSeq PE250 platform.

The samples underwent sequencing on an Illumina NovaSeq platform in accordance with the manufacturer’s guidelines. Paired-end reads were associated with their respective samples based on unique barcodes and primer sequences were removed. FLASH was utilized to merge the paired-end reads. We performed quality filtering on the raw reads using filter conditions specified in fqtrim (v0.94) to obtain high-quality clean tags. The Vsearch software (v2.3.4) was employed to remove chimeric sequences. Following dereplication using DADA2, feature tables and feature sequences were obtained. To assess alpha diversity and beta diversity, the sequences were randomly normalized. For normalization of feature abundance, we employed the SILVA database (release 138) classifier and scaled it based on the relative abundance of each sample. Alpha diversity, which measures species diversity within a sample, was evaluated using five indices: Chao1, Goods coverage, Observed species, Pielou-e, Shannon, and Simpson. The microbial structure in different groups of equine fecal samples was analyzed using principal component analysis (PCA), principal coordinate analysis (PCoA), Upgma cluster, and nonmetric multidimensional scaling (NMDS). To identify bacterial taxa that showed differential abundance across the groups, linear discriminant analysis effect size (LEfSe) was employed (32). Furthermore, the relationship between fecal microbiota and host parameters was examined using Pearson correlation analysis, performed by LC-Bio Technology.^1^ Phylogenetic Investigation of Communities by Reconstruction of Unobserved States (PICRUSt2) analysis was performed to identify bacterial functions that were altered in different groups (33).

### 2.11 Statistical analysis

GraphPad Prism 9 (San Diego, CA, United States) was used for statistical analysis. Differences between clinical and blood serum date were determined using two-way ANOVA test and the alpha diversity was calculated by using the Kruskal–Wallis test. A *p* < 0.05 is considered to be statistically significant. Statistical significance was denoted by ^*^p < 0.05, ^**^p < 0.01, ^***^p < 0.001, and ^****^p < 0.0001.

## 3 Results

### 3.1 Assessment of body temperature, fecal pH, and diarrhea score

**Figure 2:**
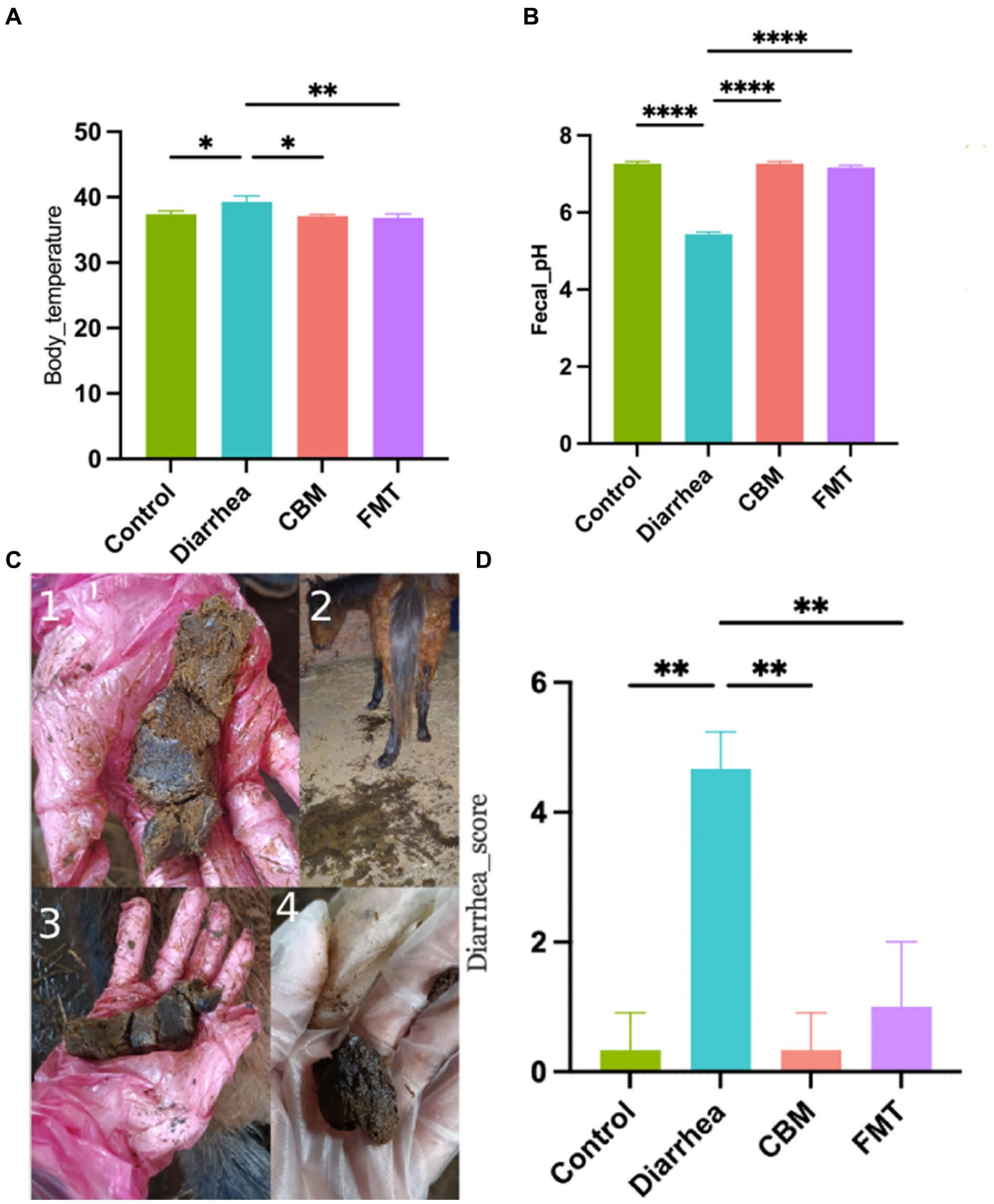
Body temperature, fecal pH, and diarrhea score in different groups. **(A)** Body temperature; **(B)** fecal pH; **(C)** fecal appearance; and **(D)** diarrhea score.

To evaluate the clinical characteristics of the horses in various groups, we measured body temperature, fecal pH, and diarrhea score in different time points (Figure 2). Body temperature and diarrhea score were significantly increased in horses with diet induced diarrhea (Control vs Diarrhea, *p* = 0.0264, *p* = 0.0023, respectively), while fecal pH significantly decreased (Control vs Diarrhea, *p* < 0.0001). After CBM and FMT treatment, body temperature and diarrhea score were significantly decreased (Diarrhea vs. CBM, *p* = 0.0134; Diarrhea vs. FMT, *p* = 0.0076); at the same time, fecal pH increased significantly and returned to normal level (Diarrhea vs. CBM, *p* < 0.0001; Diarrhea vs. FMT, *p* < 0.0001).

### 3.2 Assessment of serum levels

**Figure 3:**
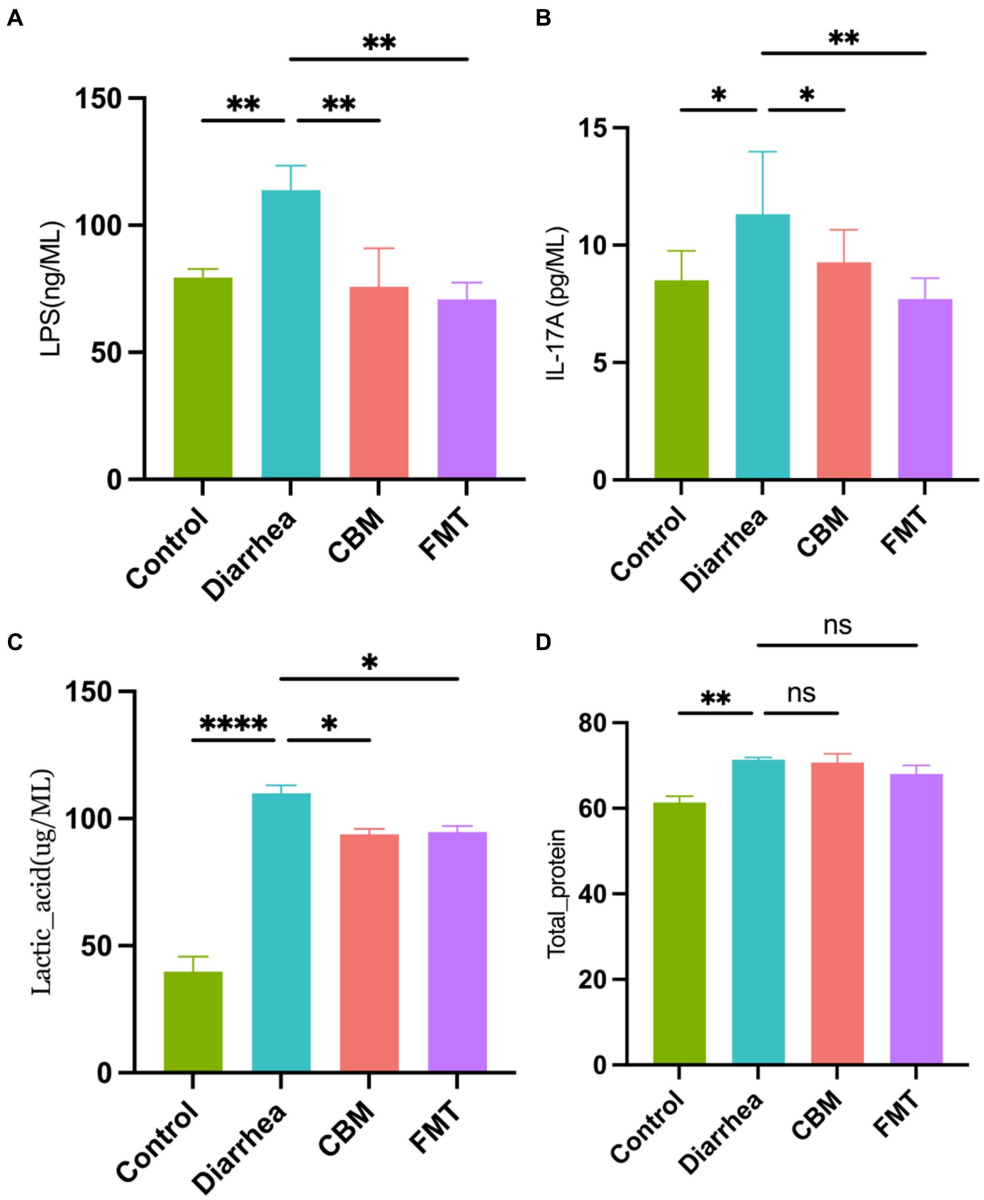
Blood serum concentrations in different groups. **(A)** LPS concentration; **(B)** IL-17A concentration; **(C)** lactic acid concentration; and **(D)** total protein concentration.

We measured LPS, IL-17A, lactic acid, and total protein levels in the serum of horses in different groups (Figure 3). Results showed that horses in Diarrhea group had systematic inflammatory reactions which were indicated by the significant increased serum levels of LPS, IL-17A, lactic acid, and total protein compared to Control group (*p* = 0.0073, *p* = 0.0117, *p* < 0.0001, *p* = 0.0019, respectively). After CBM and FMT treatment, the inflammatory indicators were significantly decrease in CBM and FMT group compared to Diarrhea group (*p* = 0.0044, *p* = 0.0023; *p* = 0.047, *p* = 0.0034; *p* = 0.0121, *p* = 0.0159), except for total protein levels (*p* = 0.965, *p* = 0.1989).

### 3.3 Assessment of intestinal tissue histopathology

**Figure 4:**
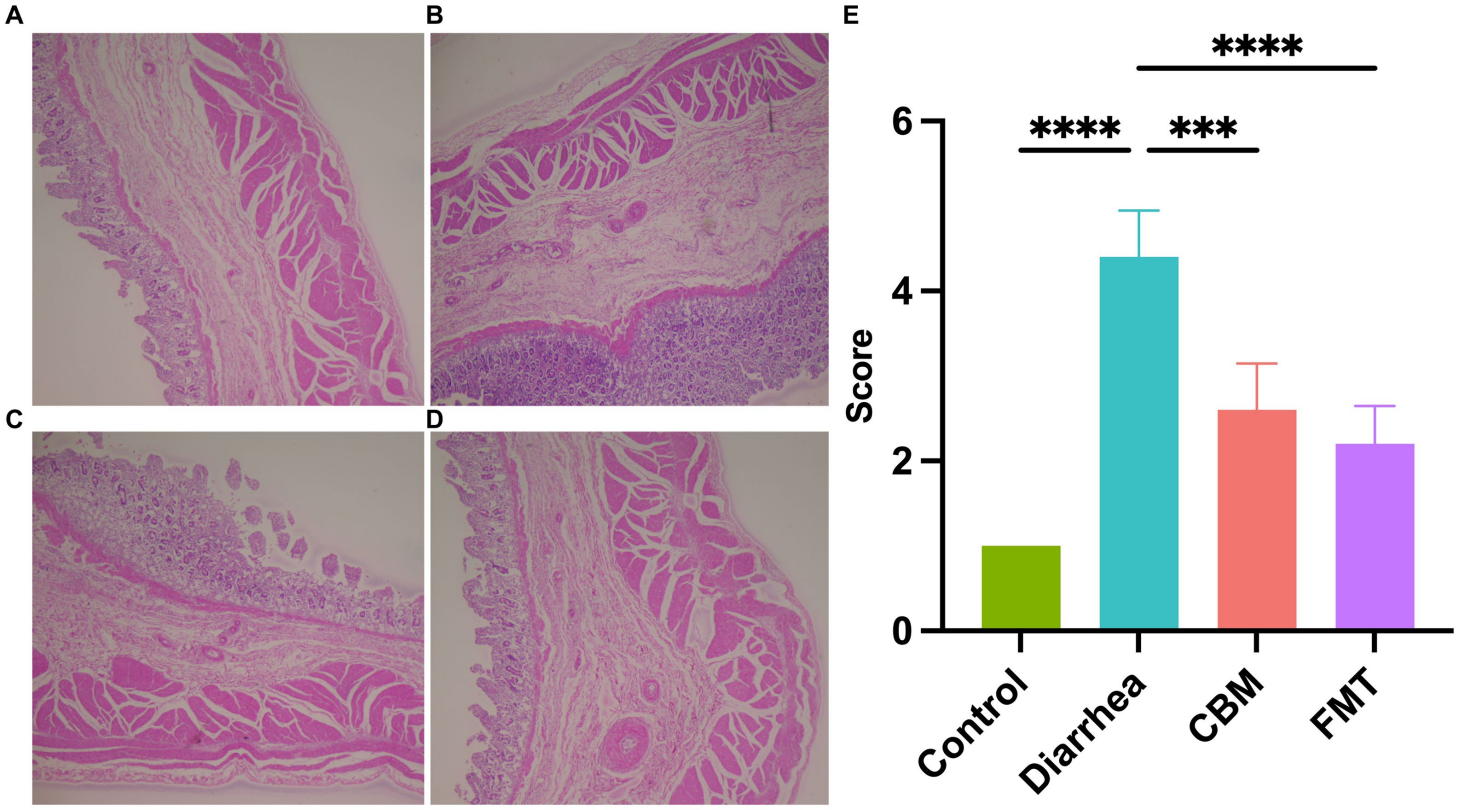
Histopathological observation of colon tissues. **(A)** Healthy horse tissue; **(B)** diarrhea horse tissue; **(C)** CBM treated horse tissue; **(D)** FMT treated horse tissue; and **(E)** scores of histopathology.

We evaluated the histopathological changes of the colon tissues of horses in different groups (Figure 4). The results showed that there are obvious differences among groups. Compared to Control group, the tissue in the Diarrhea group had damaged structural layers, inflamed mucosa, and abnormal overgrowth of cells (*p* < 0.0001). After treatment with CBM and FMT, both groups showed a reduction in tissue damages, with a more significant improvement observed in the FMT group (*p* = 0.0039; *p* < 0.0001).

### 3.4 Assessment of fecal microbiota profiles

To study the relationship between gut microbiota compositions and various health states of horses, we conducted a microbiota analysis. The results relevant to alpha diversity (Supplementary Figure S1) showed that Observed species, Shannon, Simpson, Chao1, and Pielou_e were significantly decreased in Diarrhea group compared to Control group (*p* = 0.0003; *p* < 0.0001; *p* = 0.0003; *p* < 0.0001). However, Goods coverage did not present any significant change in Diarrhea group compared to Control group (*p* = 0.0533). Following CBM treatment, Shannon, Simpson, and Pielou_e were significantly increased in Diarrhea group compared to Control group (*p* = 0.0003; *p* = 0.0002; *p* < 0.0001), while Observed species, Chao1, and Goods coverage did not show any significant changes (*p* = 0.2863; *p* = 0.2870; *p* = 0.9999). After FMT intervention, Observed species, Shannon, Simpson, Chao1, and Pielou_e were significantly increased in Diarrhea group compared to Control group (*p* = 0.0034; *p* < 0.0001; *p* = 0.0001; *p* = 0.0034; *p* < 0.0001), while and Goods coverage did not show any significant changes (*p* = 0.8031).

The beta diversity analysis (Supplementary Figure S2) revealed that the PCA (*R* = 0.5557, *p* = 0.001), PCoA (*R* = 0.6417, *p* = 0.001), Upgma cluster and NMDS plots, based on unweighted UniFrac distance, showed clear separation between Control and Diarrhea group, indicating these two groups have different microbial compositions. After CBM and FMT treatments, the gut microbiota composition changed. However, Upgma cluster plot revealed that CBM group was more similar to Diarrhea group, while FMT group was more similar to Control group. The wider range of CBM compared to FMT in PCA, PCoA, and NMDS plots also suggested there were different effects of CBM and FMT on the composition of diarrheic gut microbiota.

#### 3.4.1 Composition of the gut microbiota at the phylum level

**Figure 5:**
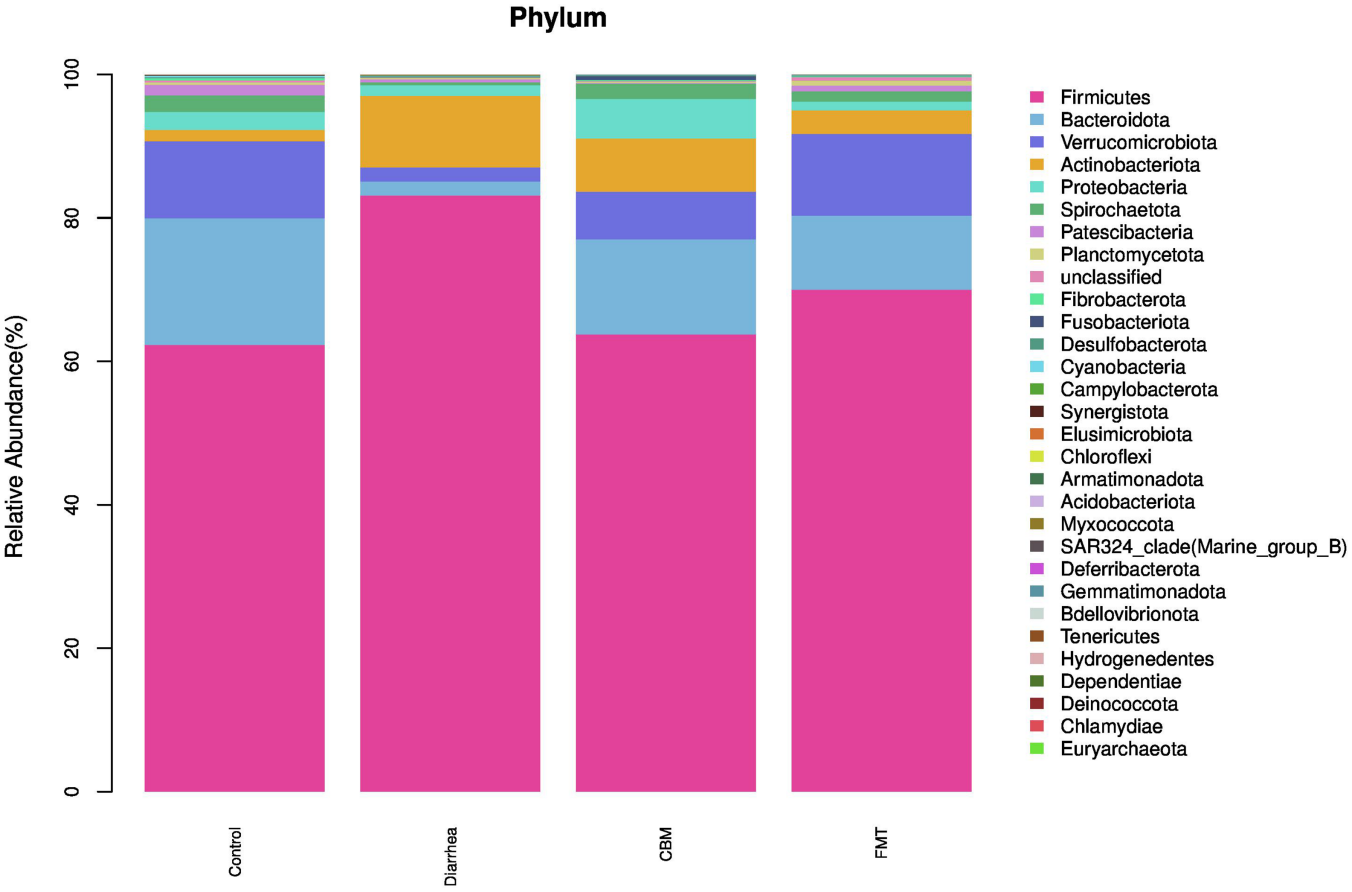
The fecal microbiota compositions in different groups at phylum level.

At the phylum level (Figure 5), *Firmicutes*, *Actinobacteriota*, and *Desulfobacterota* were increased after diarrhea induction, while *Bacteroidota*, *Verrucomicrobiota*, *Proteobacteria*, *Spirochaetota*, *Patescibacteria*, *Planctomycetota*, *Fibrobacterota*, and *Cyanobacteria* were decreased. After CBM treatment, the relative abundance of *Firmicutes*, *Actinobacteriota*, *Patescibacteria*, *Planctomycetota*, and *Cyanobacteria* were decreased, while *Bacteroidota*, *Verrucomicrobiota*, *Proteobacteria*, *Spirochaetota*, *Fibrobacterota*, and *Desulfobacterota* were increased compared to Diarrhea group. Following FMT treatment, the relative abundance of *Firmicutes*, *Actinobacteriota*, *Proteobacteria*, and *Desulfobacterota* were decreased, while *Bacteroidota*, *Verrucomicrobiota*, *Spirochaetota*, *Patescibacteria*, *Planctomycetota*, and *Fibrobacterota* were increased compared to Diarrhea group.

#### 3.4.2 Composition of the gut microbiota at the genus level

**Figure 6:**
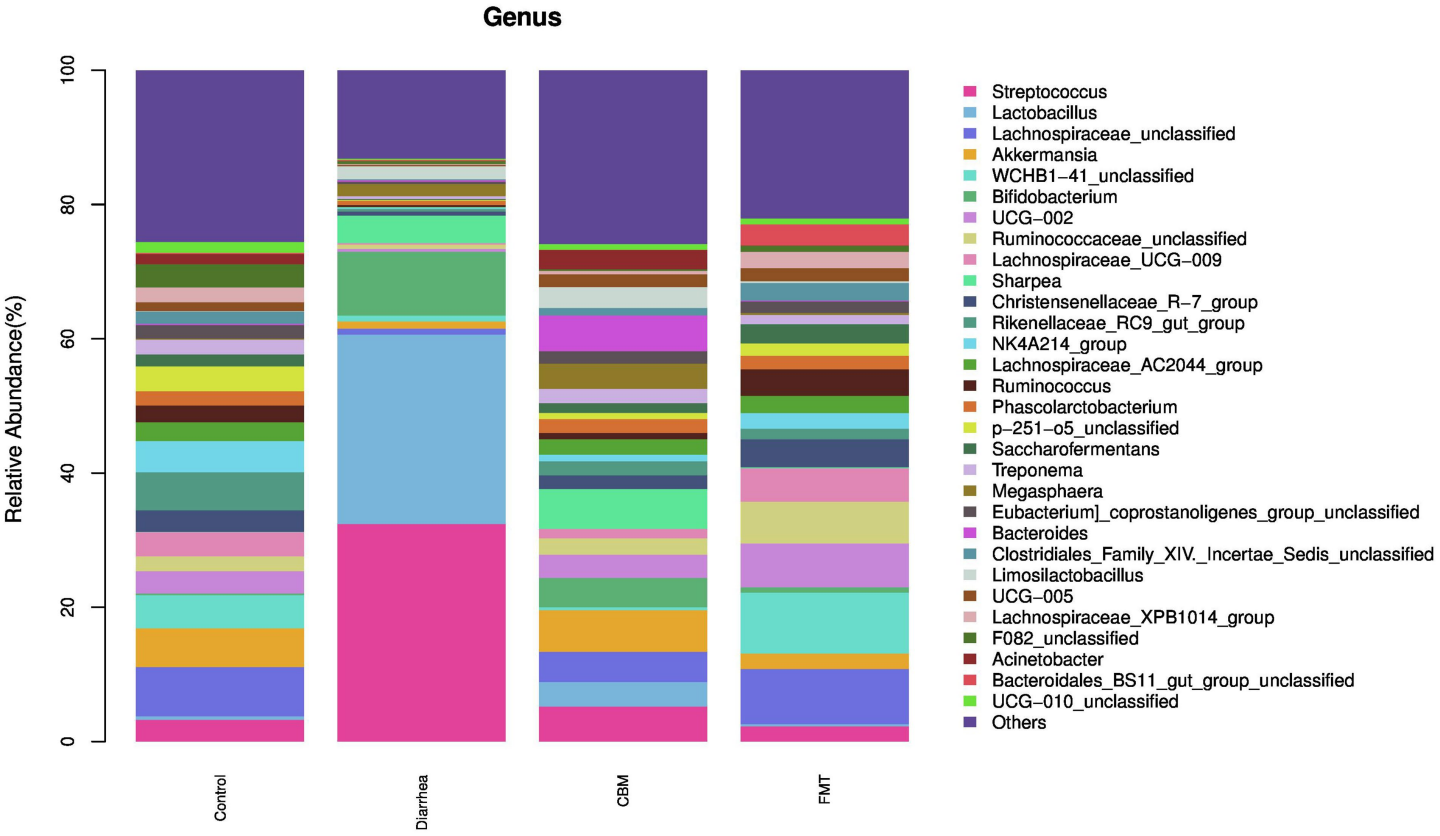
The fecal microbiota compositions in different groups at genus level.

At the genus level (Figure 6), oligofructose induced diarrhea resulted to increase relative abundance of *Streptococcus, Lactobacillus, Bifidobacterium, Sharpea, Megasphaera, Bacteroides*, and *Limosilactobacillus*, while decrease was observed in *Lachnospiraceae_unclassified, Akkermansia, UCG-002, Christensenellaceae_R_7_group, WCHB1-41_unclassified, Ruminococcaceae_unclassified, Rikenellaceae_RC9_gut_group, Lachnospiraceae_UCG-009, NK4A214_group, Lachnospiraceae_AC2044_group, Ruminococcus, Phascolarctobacterium, Saccharofermentans, Acinetobacter, Treponema, F082_unclassified, Eubacterium_coprostanoligenes_group_unclassified, Clostridiales_Family_XIV._Incertae_Sedis_unclassified, UCG-005, Lachnospiraceae_XPB1014_group, p-251-o5_unclassified, Bacteroidales_BS11_gut_group_unclassified, UCG-010_unclassified, and Others.*

CBM and FMT had a positive effect on the structure of the fecal microbiota. Especially, these treatments contributed to decrease of relative abundance of *Streptococcus*, *Lactobacillus*, and *Bifidobacterium* while resulted to increase in *Lachnospiraceae_unclassified*, *Akkermansia*, *UCG-002*, *Christensenellaceae_R_7_group*, *Ruminococcaceae_unclassified*, *Rikenellaceae_RC9_gut_group*, *Lachnospiraceae_UCG-009*, *NK4A214_group*, *Lachnospiraceae_AC2044_group*, *Ruminococcus*, *Phascolarctobacterium*, *Saccharofermentans*, *Acinetobacter*, *Treponema*, *Eubacterium_coprostanoligenes_group_unclassified*, *Clostridiales_Family_XIV_Incertae_Sedis_unclassified*, *UCG-005*, *Lachnospiraceae_XPB1014_group*, *p-251-o5_unclassified*, *UCG-010_unclassified*, and Others. Further analysis revealed that *WCHB1-41_unclassified* was increased in FMT group, but reduced further in CBM group; *Sharpea* was decreased in FMT group, but further increased in CBM group; *Megasphaera* was decreased in FMT group, but farther increased in CBM group; *Bacteroides* was decreased in FMT group, but increased farther in CBM group; *Limosilactobacillus* was decreased in FMT group, but further increased in CBM group; *Bacteroidales_BS11_gut_group_unclassified* was increased in FMT group, but further decreased in CBM group.

#### 3.4.3 Composition of the gut microbiota at the species level

**Figure 7:**
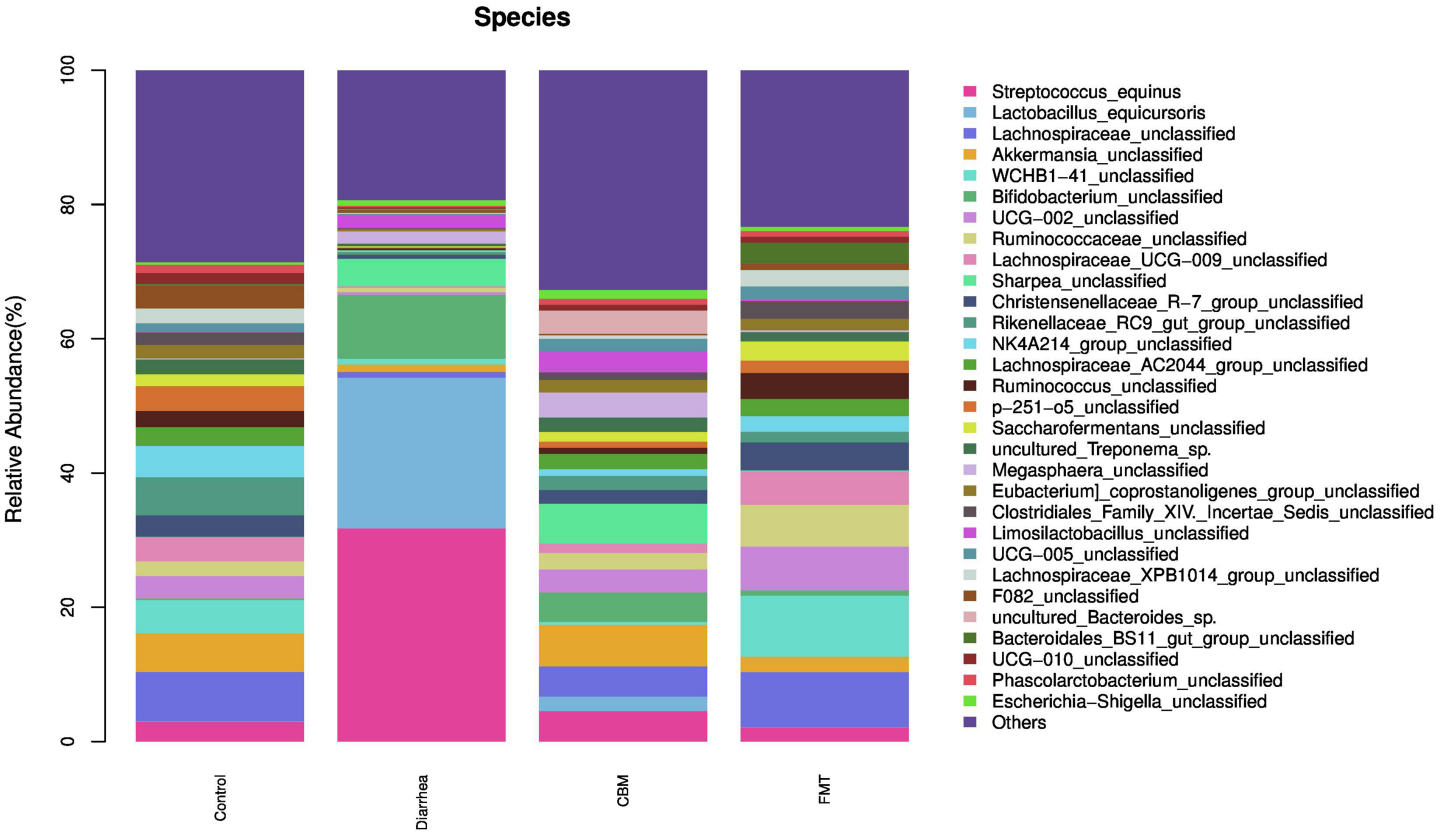
The fecal microbiota compositions in different groups at species level.

At the species level (Figure 7), oligofructose induced diarrhea resulted to increase relative abundance of *Streptococcus_equinus*, *Lactobacillus_equicursoris*, *Bifidobacterium_unclassified*, *Sharpea_unclassified*, *Megasphaera_unclassified*, *Limosilactobacillus_unclassified*, *uncultured_Bacteroides_*sp., and *Phascolarctobacterium_unclassified*. After FMT treatment, *Streptococcus_equinus*, *Lactobacillus_equicursoris*, *Bifidobacterium_unclassified*, *Sharpea_unclassified*, *Megasphaera_unclassified*, and *Limosilactobacillus_unclassified* were decreased in FMT group compared to Diarrhea group. Following CBM treatment, *Streptococcus_equinus*, *Lactobacillus_equicursoris*, and *Bifidobacterium_unclassified* were decreased, while *Sharpea_unclassified*, *Megasphaera_unclassified*, and *Limosilactobacillus_unclassified* were further increased in CBM group compared to Diarrhea group.

We further conducted LEfSe analysis to identify the bacterial taxa that were changed in the various groups (Supplementary Figure S3). The results showed that there were 14 taxa decreased, and 8 taxa enriched, while 27 taxa unchanged in Diarrhea group compared to Control group. After CBM treatment, there were 4 taxa decreased, and 18 taxa increased, while 27 taxa remained unaffected in CBM group compared to Diarrhea group. After FMT treatment, 6 taxa were decreased, and 10 taxa increased, while 32 taxa remained unchanged in FMT group compared to Diarrhea group.

**Figure 8:**
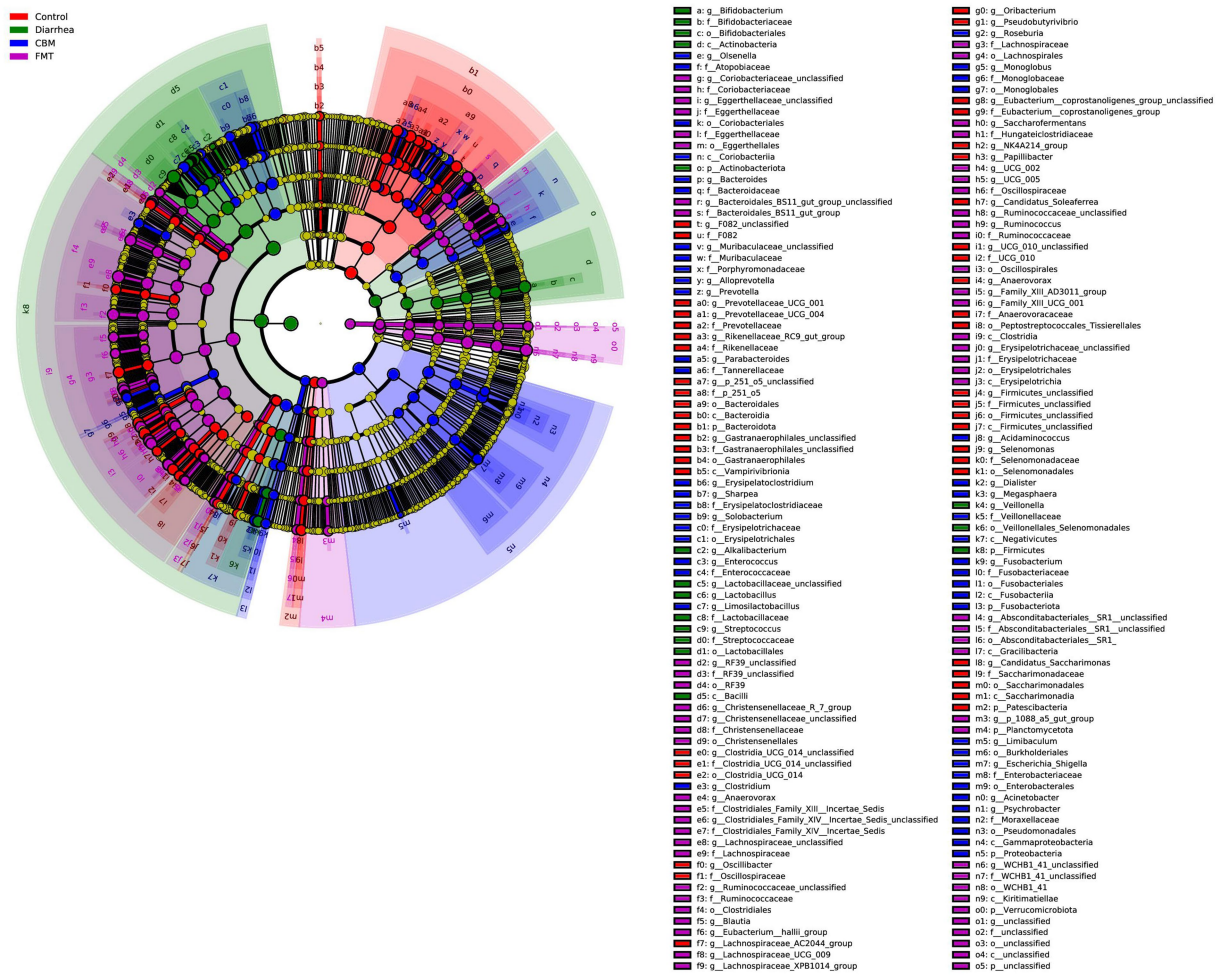
Cladogram generated from LEfSe analysis showing the relationship between taxon at the levels of phylum, class, order, family, genus, and species.

The Cladogram created from LEfSe analysis showed the relationship between taxon at the levels of phylum, class, order, family, and genus (Figure 8). Results showed that, in Diarrhea group, at the genus level, the biomarkers with significant discriminative power were *Bifidobacterium*, *Alkalibacterium*, *Lactobacillaceae_unclassified, Lactobacillus, Streptococcus,* and *Veillonella*; in CBM group, the biomarkers were *lsenella, Bacteroides, Muribaculaceae_unclassified, Alloprevotella, Prevotella, Parabacteroides, Erysipelatoclostridium, Sharpea, Solobacterium, Limosilactobacillus, Clostridium, Roseburia, Monoglobus, Acidaminococcus, Dialister, Megasphaera, Fusobacterium, Limibaculum, Escherichia_Shigella, Acinetobacter*, and *Psychrobacter*; in FMT group, the biomarkers were *Coriobacteriaceae_unclassified, Eggerthellaceae_unclassified, Bacteroidales_BS11_gut_group_unclassified, RF39_unclassified, Christensenellaceae_R_7_group, Christensenellaceae_unclassified, Anaerovorax, Clostridiales_Family_XIV_Incertae_Sedis_unclassified, Lachnospiraceae_unclassified, Ruminococcaceae_unclassified, Blautia, Eubacterium_hallii_group, Lachnospiraceae_UCG_009, Lachnospiraceae_XPB1014_group, Saccharofermentans, UCG_002, UCG_005, Ruminococcaceae_unclassified, Ruminococcus, Family_XIII_AD3011_group, Family_XIII_UCG_001, Erysipelotrichaceae_unclassified, Absconditabacteriales_SR1_unclassified, p_1,088_a5_gut_group*, and *WCHB1_41_unclassified*.

#### 3.4.4 Correlation between host clinical and serum parameters and key bacterial genera

**Figure 9:**
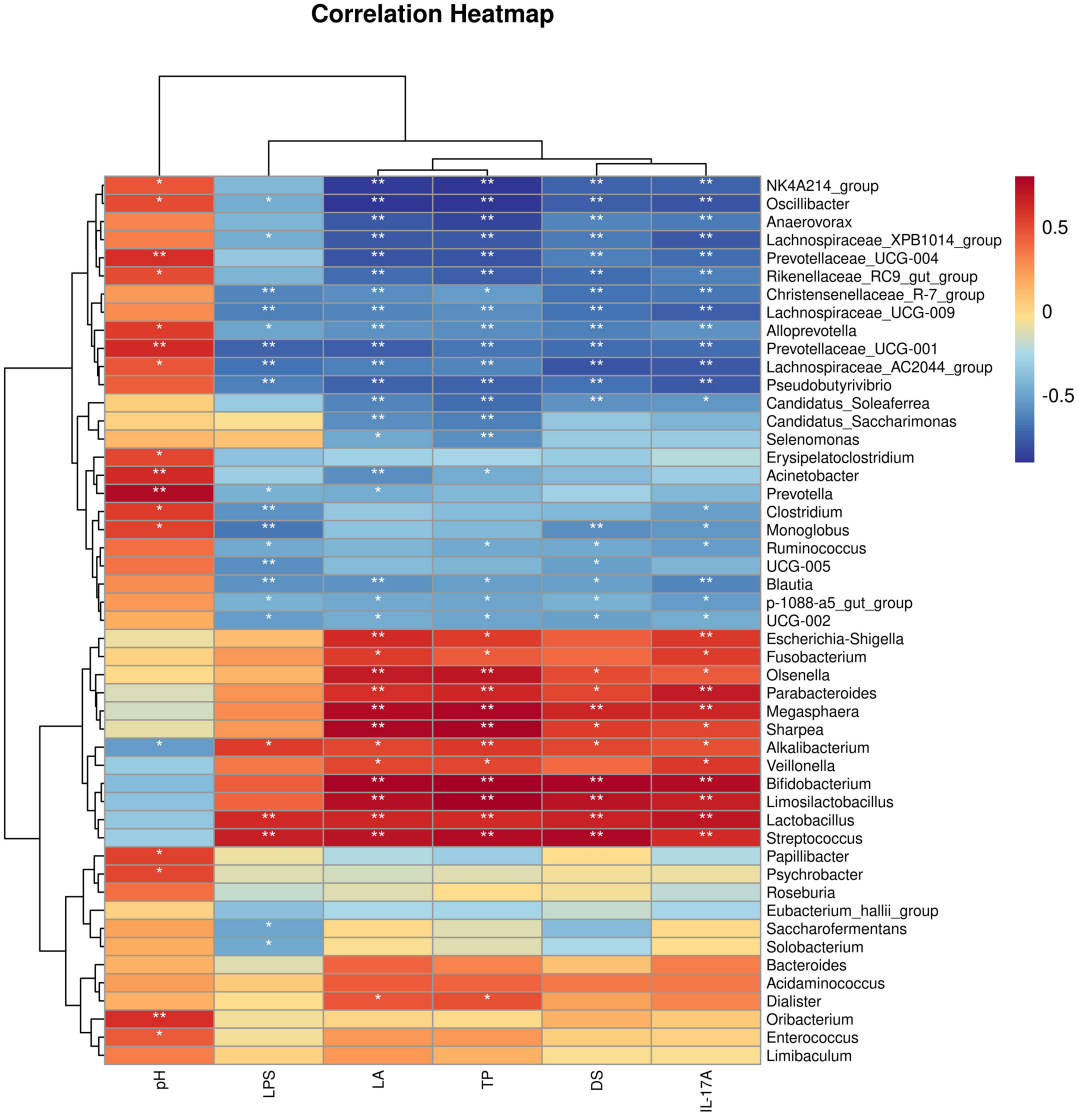
Correlation heatmap between bacterial genera and host clinical and serum parameters.

Then, we analyzed the relationships between host clinical and serum parameters and key bacterial genera identified by LEfSe in different groups (Figure 9). Our analysis revealed significant positive correlations between *Alkalibacterium*, *Lactobacillus*, and *Streptococcus* were significantly positively correlated with LPS, Lactic acid, Total protein, Diarrhea score, and IL-17A, and *Alkalibacterium* was significantly negatively correlated with pH levels. In addition, *Lachnospiraceae_XPB1014_group, Christensenellaceae_R_7_group, Lachnospiraceae_UCG_009, Alloprevotella, Prevotellaceae_UCG_001, Lachnospiraceae_AC2044_group, Pseudobutyrivibrio, Blautia, p_1088_a5_gut_group*, and *UCG_002* were significantly negatively correlated with LPS, Lactic acid, Total protein, Diarrhea score, and IL-17A, while *Oscillibacter, Alloprevotella, Prevotellaceae_UCG_001*, and *Lachnospiraceae_AC2044_group* were significantly positively correlated with pH levels at the same time.

#### 3.4.5 Predicted functional profiles of the gut microbiota community

The functional profiles of the gut bacterial community were predicted using PICRUSt2, and the results identified a total of 15 gene families in all samples at the second tier (Supplementary Figure S4). Among the predicted KEGG pathways, most of the sequences were assigned to gene families carbohydrate metabolism, amino acid metabolism, energy metabolism, Metabolism of Cofactors and Vitamins, Enzyme Families, Cell Motility, Metabolism, and Signal Transduction.

**Figure 10:**
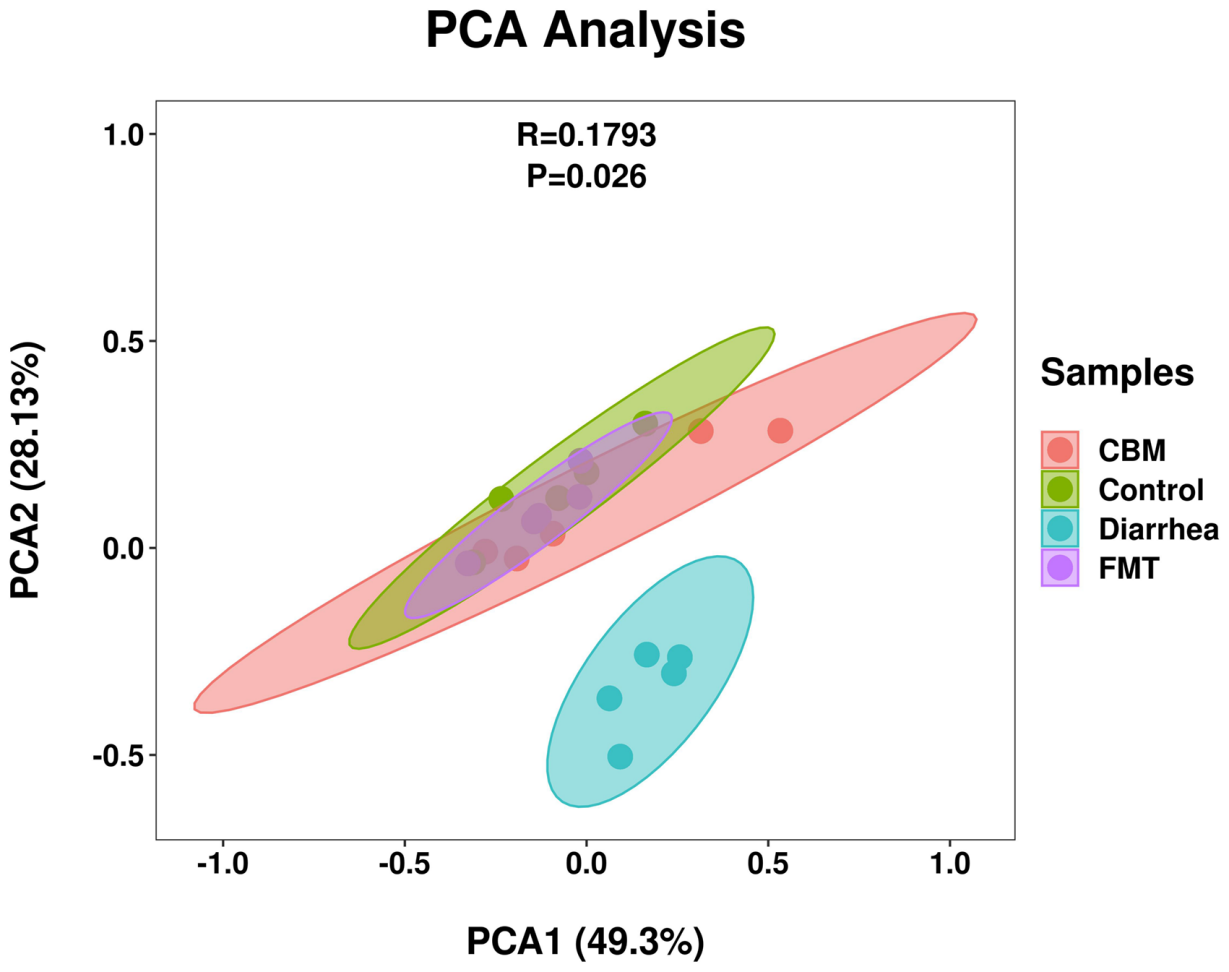
PCA plot of gut bacterial functions in different groups.

To further explore the implications of the gut bacterial functions, PCA was conducted and results showed that Control, CBM, and FMT group samples gathered together, and separated from Diarrhea group samples (Figure 10). This separation was supported by ANOSIM analysis (*R* = 0.1793, *p* = 0.026), which suggested a significant difference across different groups.

## 4 Discussion

Diarrhea is a common health issue in horses that can substantially impact their overall well-being and performance. Recognizing the significance of this condition is crucial, as it not only causes discomfort and distress to affect horses but also poses potential economic and management challenges for horse owners and caretakers. In horses, diarrhea is a multifactor-caused condition influenced by various factors. These factors include infectious agents such as bacteria, viruses, parasites, and protozoa (34–36), diseases like inflammatory bowel disease and colitis (37, 38), as well as other factors such as dietary changes, stress, and antimicrobial therapy (30, 39). However, among these various factors, dysbiosis of the gastrointestinal microbiota emerges as a crucial underlying mechanism driving the onset of diarrhea (40). Therefore, restoring disbalanced gut microbial community may be an effective approach for treating diarrhea in horses.

To explore the manipulation of gut microbiota as a potential treatment option for diarrhea in horses, previous studies have investigated fecal microbiota transplantation (FMT). These studies have identified greater beta diversity and increased abundance of genera such as *Lactobacillus*, *Intestinimonas*, and *Streptococcus* in horses with diarrhea prior to FMT (19). Following FMT treatment, horses displayed higher alpha diversity and a lower mean UniFrac distance (similar to the donor fecal microbiota) (17, 18), indicating positive outcomes. However, one study reported no significant changes after conducting FMT (19). Furthermore, additional studies have also indicated the ineffectiveness of FMT in horses for the treatment of diarrhea. One particular study found no impact of FMT in addressing free fecal water in horses, suggesting that gut microbiota alterations may not be a contributing factor to this condition (20). Another study revealed that FMT achieved only a 67% success rate in managing diarrhea in a small cohort of six horses (19). Moreover, FMT was found to be ineffective in preventing metronidazole-induced gut microbiota dysbiosis in horses in another study (21). However, it is worth noting that this particular study utilized a relatively small amount of stool (500 g) for the transplantation, which may not have been sufficient to induce significant changes in the gastrointestinal tract of the horse. Additionally, a study involving 111 horses reported no effectiveness of FMT in their cohort (22). However, it is important to acknowledge that in this study, the authors failed to exclude horses treated with antimicrobials from the statistical analysis when evaluating the efficacy of FMT. These findings underscore the importance of ongoing research to elucidate the role of FMT in equine health.

In addition, our observations, along with previous research conducted on both dairy cows and through *in vitro* studies (41–43), have demonstrated the beneficial effects of sodium bicarbonate buffer in modulating the gastrointestinal environment. These studies consistently indicate that sodium bicarbonate exhibits advantageous properties such as raising pH levels, reducing lactate and biogenic amine concentrations, mitigating rumen acidosis, and enhancing dry matter intake. Based on these, we hypothesized that CBM, which mainly consists of sodium bicarbonate (formula shown in Table 2), may positively regulate equine gastrointestinal environment and alleviate diarrhea.

In this paper, we established an oligofructose-induced diarrhea model as the basis of our study. This choice stems from the prevalence of cases in clinical settings where we observed diarrhea in horses primarily associated with dietary changes and antibiotic therapy, rather than pathogen-related causes. Given that these horses were intended for meat production, we opted to use oligofructose instead of antibiotics. Furthermore, in modern times, horses are often provided with higher concentration diets to enhance their performance. By employing this model in a controlled manner, our study aims to investigate various treatment options for diarrhea in horses, thus providing valuable insights not only into the management of diarrhea but also pertaining to the specific treatment approaches applied to affected horses. Through this research, we anticipate shedding light on effective strategies for addressing and alleviating diarrhea in equine populations.

The results demonstrated that induction of diarrhea negatively impacted horses including body temperature, fecal pH, and diarrhea score. These trends mirror those observed in natural or antibiotic-induced diarrhea cases in horses (18, 44), suggesting the oligofructose-induced model could serve as a valuable investigative tool for studying equine diarrhea. This approach avoids the risks associated with antibiotic use, which can elicit severe consequences in horses such as colitis (45–48), diarrhea (49, 50), colic (51), and laminitis (52). Following CBM and FMT treatments, the diarrhea-associated clinical symptoms significantly improved in horses from both groups. These findings indicate CBM and FMT may be promising therapeutic approaches for treating diarrhea in horses.

Next, we conducted an examination of blood serum levels within different groups to investigate the impact of diarrhea on horses. The results unveiled a notable increase in systemic inflammation in horses with diarrhea, reflected by elevated quantities of cytokines in the serum. Specifically, concentrations of LPS, IL-17A, total protein, and lactic acid were significantly higher in the serum of horses in Diarrhea compared to Control group. These results point towards an active immune response and a disrupted homeostatic balance, suggesting diarrhea triggers an enhanced inflammatory response throughout the body in affected horses. To evaluate potential approaches, we assessed the effects of CBM and FMT treatments. After the administration of CBM and FMT, considerable reductions in the levels of LPS, IL-17A, and lactic acid were observed. This suggests that both CBM and FMT hold promise as viable tactics for attenuating the inflammatory response associated with diarrhea in horses. The significant decrease in the concentration of LPS in the treated group indicates less endotoxin release. The significant decrease in the concentration of lactic acid-a biomarker of tissue perfusion, in the treated groups indicating the activation of the anaerobic metabolism pathway (53). These may indicate a beneficial impact on the overall burden. However, there was no significant change in total protein levels following CBM and FMT treatments. This implies that while these interventions effectively address specific aspects of the inflammatory response, they may not have a direct influence on total protein concentrations in the serum. These findings shed light on the complex interplay between diarrhea, inflammation, and potential therapeutic interventions in horses.

Moreover, we examined the histopathology of the colon tissue to identify differences among the various treatment groups. The Diarrhea group displayed evident structural damages, overgrowth of cells, and inflamed mucosa compared to the Control group. Following CBM and FMT treatments, both CBM and FMT groups exhibited reduced inflammation. Notably, FMT appeared more effective in repairing tissue architecture, suppressing abnormal cell proliferation, and restoring intestinal homeostasis compared to CBM. While both treatments displayed anti-inflammatory effects, FMT demonstrated superior efficacy in reversing pathologic changes associated with diarrhea. These findings further underscore the potential of manipulating the gastrointestinal environment and/or microbial composition as effective approaches for treating diarrhea in horses.

Furthermore, in the present study, we found that the richness and diversity of fecal bacterial community were decreased significantly after the occurrence of diarrhea, which evidenced by analyzing chao1, observed species, Shannon, Simpson, Goods coverage and Pielou_e. Following CBM and FMT treatments, there were significant alterations in the gut microbiota composition, particularly in terms of alpha diversity (except for Goods-coverage), with FMT demonstrating a more pronounced and impactful effect. Regarding beta diversity, the composition of fecal microbiota in Control and Diarrhea group horses were significantly different, a clear separation was observed both in PCA, PCoA, Upgma cluster, and NMDS plots. After FMT treatment, a notable distinction in the fecal microbiota composition between the Diarrhea and FMT groups of horses became evident. This differentiation was clearly observed in various analytical plots, including PCA, PCoA, Upgma cluster, and NMDS. Furthermore, the FMT intervention induced a transformation in the gut microbiota composition of the diarrhea-afflicted horses, aligning it more closely with that of healthy horses. In contrast, following the administration of CBM, the gut microbiota composition in the CBM group exhibited a closer resemblance to that of Diarrhea group rather than Control group. This distinction was particularly conspicuous in the Upgma cluster plot, where the CBM samples clustered together with the diarrhea samples. These findings strongly indicate that FMT outperforms CBM in terms of restoring dysbiosis in the gut microbiota and promoting a healthier composition.

We, then, investigated the compositional differences of the gut microbiota across different groups. At phylum level, *Firmicutes, Actinobacteriota, Desulfobacterota*, and *Fusobacteriota* were increased significantly, while *Bacteroidota, Patescibacteria, Armatimonadota, Cyanobacteria, Verrucomicrobiota, SAR324_clade (Marine_group_B), Proteobacteria, and Planctomycetota* were significantly decreased in Diarrhea group compared to Control group. Following CBM and FMT treatments, *Firmicutes, Bacteroidota, and Actinobacteriota* were changed similarly in both CBM and FMT groups with more towards Control group. However, *Patescibacteria and Planctomycetota* were further decreased in CBM group while increased in FMT group compared to Diarrhea group; *Proteobacteria, Desulfobacterota*, and *Fusobacteriota* were further increased in CBM group while decreased in FMT group compared to Diarrhea group.

At genus level, *Streptococcus, Lactobacillus, Bifidobacterium, Sharpea, Limosilactobacillus*, and *Megasphaera* were significantly enriched, while *Akkermansia* and *Lachnospiraceae_UCG-009* were significantly decreased in Diarrhea group compared to Control group. Following CBM and FMT treatments, diarrhea-leading genus, *Streptococcus* and *Lactobacillus* were significantly decreased in both groups compared to Diarrhea group. However, it’s worth noting that the relative abundances of these genera remained higher in the CBM group when compared to the Control and FMT groups. This observation provides a plausible explanation for the closer resemblance of the gut microbiota compositions in the CBM group to those of the Diarrhea group.

At the species level, *Streptococcus_equinus*, *Lactobacillus_equicursoris*, *Bifidobacterium_unclassified*, *Sharpea_unclassified*, *Megasphaera_unclassified*, and *Limosilactobacillus_unclassified* were increased significantly in Diarrhea group compared to Control group. Following CBM and FMT treatments, the relative abundance of *Streptococcus_equinus*, *Lactobacillus_equicursoris* and *Bifidobacterium_unclassified* were decreased significantly in both groups compared to Diarrhea group, however, in CBM group these species were higher compared to Control group. In addition, *Sharpea_unclassified*, *Megasphaera_unclassified*, and *Limosilactobacillus_unclassified* were significantly decreased in FMT group but showed further increases in in CBM group.

These findings suggest that CBM and FMT may operate through distinct mechanisms in restoring gut microbiota dysbiosis induced by diarrhea. Nevertheless, both CBM and FMT treatments effectively alleviated diarrhea-related symptoms in horses, suggesting the involvement of additional factors, possibly metabolomic changes, in their therapeutic mechanisms. Additionally, we observed an increase in pH levels in both CBM and FMT groups. This pH elevation could be attributed to the reduction in Lactobacillus levels in the FMT group, while in CBM group, it may be due to chemical compounds rather than bacterial alterations.

We conducted a detailed investigation to assess the potential link between changes in the gut microbiota and clinical and blood serum data collected from different groups of horses. This analysis was based on Spearman correlations using bacterial genera identified through LEfSe analysis. Our findings revealed a significant association between increased bacterial genera and diarrhea, particularly key biomarker-genera such as *Bifidobacterium, Alkalibacterium, Lactobacillus, Streptococcus*, and *Veillonella*. These genera exhibited a positive correlation with deteriorated clinical parameters in the host and intensified systemic inflammatory responses. Furthermore, we observed that alterations in bacterial genera following CBM and FMT treatments, especially noteworthy biomarkers like *Alloprevotella, Monoglobus, Acinetobacter, Christensenellaceae_R_7_group, Anaerovorax, Blautia, Lachnospiraceae_UCG_009, Lachnospiraceae_XPB1014_group*, and *UCG_002*, were negatively correlated with diarrhea-related clinical symptoms and host systemic inflammatory responses. This implies that the gut microbiota plays a vital role in both the development and treatment of diarrhea induced by oligofructose in horses, highlighting its significance in the context of equine health.

Finally, we conducted a PICRUSt2 analysis. The results suggested that CBM and FMT treatments not only restore the compositions of the disrupted gut microbiota, but also reactivate the functional integrity of the bacterial communities within the gut.

These findings provide compelling evidence for the positive therapeutic effects of CBM and FMT treatments in alleviating clinical symptoms, suppressing proinflammatory cytokines, repairing tissue damages, and restoring dysbiosis gut microbiota compositions as well as its functions. Particularly intriguing, our observations reveal notable differences between the CBM and FMT groups in their ability to reestablish the gut microbiota in horses with induced diarrhea, suggesting divergent therapeutic mechanisms at play. While the precise mechanisms are yet to be fully understood, we propose a hypothesis regarding the primary objective of fecal microbiota transplantation (FMT) in the context of reconstructing a normal gut microbiota composition. This process is believed to involve niche exclusion, increased competition for nutrition, production of antimicrobials, and elevated production of secondary bile acids (54). On the other hand, CBM is postulated to create a favorable environment for the proliferation of beneficial bacterial strains, thereby promoting a healthier gut microbiota profile. Based on these findings, we propose a comprehensive approach to treat horses with diarrhea, involving the combined use of CBM and FMT treatments. By administering CBM initially to create a favorable survival environment for beneficial bacterial taxa, followed by FMT to restore disrupted microbial communities, we can optimize the therapeutic outcomes for equine diarrhea management.

## 5 Conclusion

Diarrhea poses a significant health and performance challenge for horses, with its multifactorial nature requiring effective interventions. Our study focused on the restoration of a balanced gut microbiota using FMT and CBM therapies. Notably, both treatments demonstrated substantial improvements in clinical symptoms, inflammation, and tissue damage associated with diarrhea. We observed discernable alterations in the composition of the gut microbiota during diarrhea, characterized by reduced diversity and specific changes in bacterial genera. Correlation analysis further highlighted the potential involvement of certain bacterial genera in systemic inflammatory responses and their associations with host clinical parameters. Importantly, both CBM and FMT treatments induced significant changes in the composition of the gut microbiota. These changes were accompanied by improvements in host clinical parameters and reductions in inflammation in both treatment groups. To summarize, our study provides valuable insights into the management and treatment of diarrhea in horses. The promising outcomes of CBM and FMT therapies and their ability to ameliorate clinical symptoms and restore dysbiosis of the gut microbiota associated with diarrhea offer potential avenues for effective interventions.

## Data availability statement

The original contributions presented in the study are publicly available. This data can be found at: https://www.ncbi.nlm.nih.gov/bioproject/; PRJNA1003028.

## Ethics statement

The animal study was approved by the Institutional Animal Care and Use Committee (IACUC) of Jilin University Ethics Committee. The study was conducted in accordance with the local legislation and institutional requirements.

## Author contributions

MT: Writing – original draft, Writing – review & editing. RT: Data curation, Writing – review & editing. XH: Methodology, Writing – review & editing. YF: Project administration, Writing – review & editing. NZ: Conceptualization, Writing – review & editing.

## Footnotes

^1^ https://www.omicstudio.cn/

## Supplementary material

Supplementary FIGURE S1 Alpha diversity of gut microbiota in different groups of horses. **A**, Observed species (Kruskal-Wallis, *P* = 0.0014); **B**, Shannon (Kruskal-Wallis, *P* = 0.00087); **C**, Simpson (Kruskal-Wallis, *P* = 0.0019); **D**, Chao1 (Kruskal-Wallis, *P* = 0.0014); **E**, Goods coverage (Kruskal-Wallis, *P* = 0.73); and **F**, Pielou_e (Kruskal-Wallis, *P*=0.0013).

Supplementary FIGURE S2 Beta diversity of gut microbiota in different groups of horses. **A**, PCA plot; **B**, PCoA plot; **C**, Upgma cluster plot; and **D**, NMDS plot.

Supplementary FIGURE S3 LEfSe analysis showing the relationship between taxon at the levels of phylum, class, order, family, genus, and species.

Supplementary FIGURE S4 PICRUSt2 plot of different groups.
