# Supplementary figures and images for: Carbonate buffer mixture and fecal microbiota transplantation hold promising therapeutic effects on oligofructose-induced diarrhea in horses
Source: Front Vet Sci. 2024 Apr 22;11:1388227. doi: 10.3389/fvets.2024.1388227 (PMC11071171; doi:10.3389/fvets.2024.1388227)

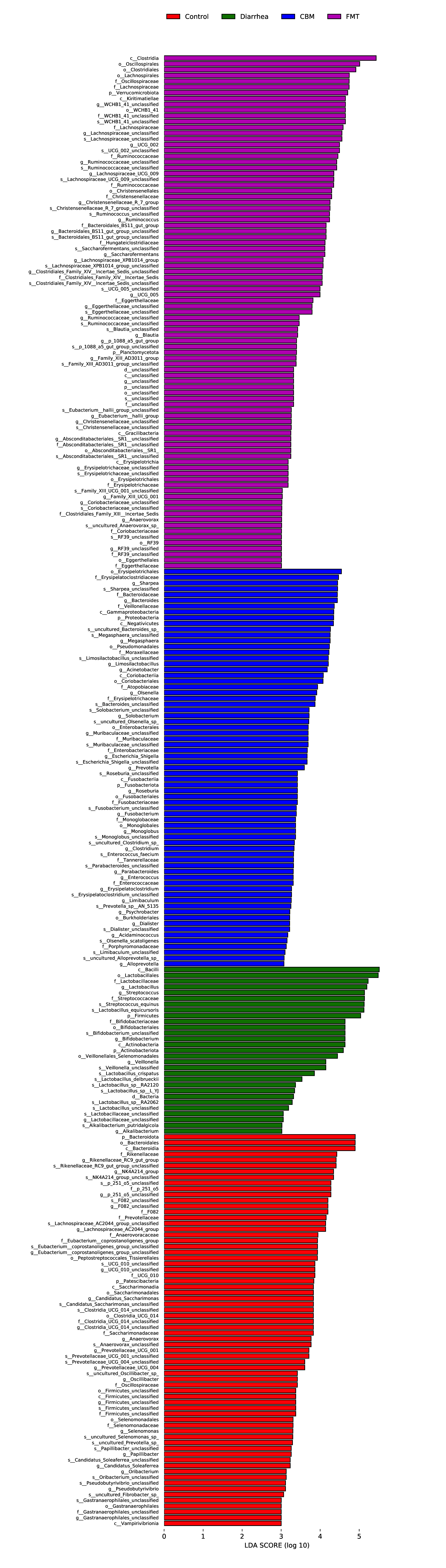

Supplement: Supplementary FIGURE S3 — LEfSe analysis showing the relationship between taxon at the levels of phylum, class, order, family, genus, and species. [file Image_3.PNG]

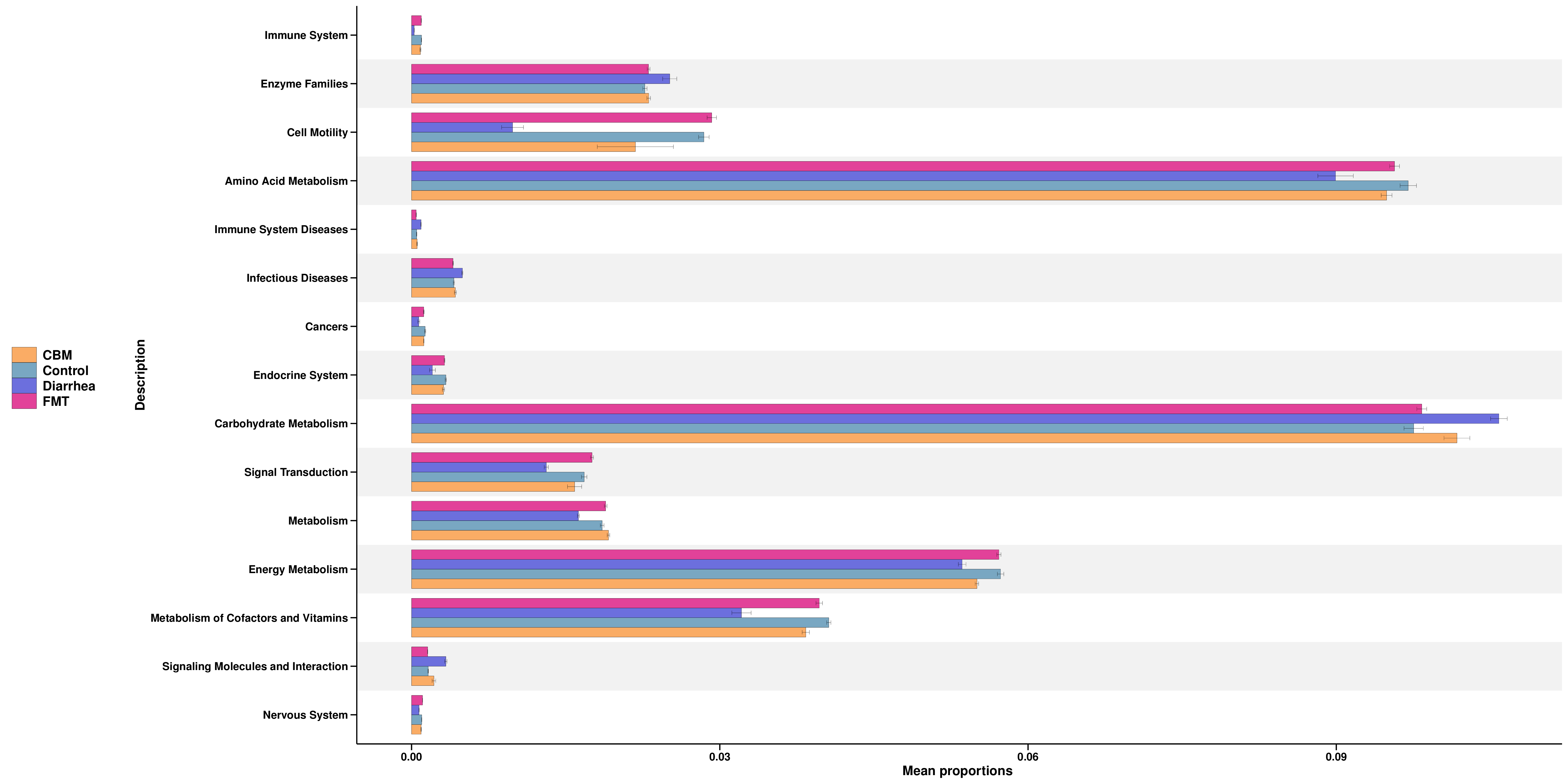

Supplement: Supplementary FIGURE S4 — PICRUSt2 plot of different groups. [file Image_4.PNG]
